# Psychological Distress Affects Performance during Exercise-Based Cardiac Rehabilitation

**DOI:** 10.3390/life14020236

**Published:** 2024-02-08

**Authors:** Marta Ricci, Gino Pozzi, Naike Caraglia, Daniela P. R. Chieffo, Daniela Polese, Leonarda Galiuto

**Affiliations:** 1 Department of Clinical and Molecular Medicine, “Sapienza” University of Rome, 00189 Rome, Italy; ricci.1583613@studenti.uniroma1.it; 2 UOC of Cardiology, Sant’Andrea University Hospital, 00189 Rome, Italy; 3 Department of Psychiatry, Fondazione Policlinico A. Gemelli-IRCCS, Catholic University, 00153 Rome, Italy; gino.pozzi@unicatt.it; 4 Clinical Psychology Unit, Fondazione Policlinico A. Gemelli-IRCCS, Università Cattolica del Sacro Cuore, 00168 Rome, Italy; naike.caraglia@policlinicogemelli.it (N.C.); danielapiarosaria.chieffo@unicatt.it (D.P.R.C.); 5 Memory Clinic, Foundation Policlinico A. Gemelli-IRCCS, 00168 Rome, Italy; 6 UOD of Childhood Neuropsychiatry, Sant’Andrea University Hospital, “Sapienza” University of Rome, 00189 Rome, Italy; daniela.polese@uniroma1.it; 7 Department of Neuroscience, Mental Health and Sensory Organs NESMOS, “Sapienza” University of Rome, 00185 Rome, Italy

**Keywords:** cardiac rehabilitation, psychological distress, depression, anxiety

## Abstract

Background: It is known that psychosocial distress affects the morbidity and mortality of patients with cardiovascular disease of every age. The aim of this study was to produce novel information on how psychological distress can influence cardiovascular performance in patients after cardiac surgery undergoing multidisciplinary cardiac rehabilitation. Methods: Patients (n = 57) admitted after cardiac surgery for valvular or coronary disease underwent, within 5 days of admission, the Symptom Checklist-90-Revised (SCL-90-R) self-report questionnaire to measure psychiatric symptoms and the 12-item General Health Questionnaire (GHQ-12) to assess the level of psychological distress. The Positive Symptom Distress Index (PSDI) was measured to indicate the amplitude of symptom distress. Cardiovascular performance was assessed by a 6 min walking test (6MWT) at admission and discharge, and oxygen consumption (VO2 max) was derived. Results: Within the SCL-90-R score, somatic symptoms (47.4%), depressive and anxiety symptoms (36.8% and 33.3%, respectively), symptoms of phobic anxiety (21.1%), and psychoticism (24.6%) were over-represented. As for the GHQ-12, 75.4% of the sample reported an abnormally negative perception of their health status. An inverse correlation was shown between the variation in 6MWT and SCL depression (*p* = 0.048), PSDI (*p* = 0.022), and the GHQ-12 (*p* = 0.040). Similarly, an inverse correlation was shown between the variation in the VO2 max, GHQ-12 (*p* = 0.041), and the PSDI (*p* = 0.023). Conclusions: Post-cardiac surgery cardiac rehabilitation was associated with increased symptoms of psychological discomfort, as compared with the general population. The amplitude of psychological distress, depression, and hostility are associated with limited improvement in performance. These data strengthen the need for psychological support during cardiac rehabilitation programs.

## 1. Introduction

Psychological distress is a widespread element of the human experience. Moreover, each individual’s response to psychological stress is subjective and determines different health consequences [1]. Although still considered a transient difficulty of little relevance, chronic stress has significant pathological consequences, including cardiovascular disease. The mechanisms by which psychological distress acts in the development of cardiovascular disease are varied; chronic stress can in fact act as an independent cardiovascular risk factor or as an amplifier of the most known cardiovascular risk factors. Particularly, psychological distress increases the risk of developing diabetes and worsens glycemic control among diabetics [2], and stress correlates with increased adiposity [3] and hypertension [4]. Furthermore, chronic stress favors smoking [5] and other unhealthy habits. There is evidence in the literature underlining how psychosocial factors affect the morbidity and mortality of patients with known cardiovascular disease, particularly regarding those with known coronary heart disease [6]. The Interheart study was an international case–control study that assessed the relationship between modifiable risk factors and coronary heart disease in 24,767 patients [7]. This study evidenced that psychological distress was correlated with an increased risk for myocardial infarction and this association was consistently represented in all ethnic groups, ages, and sexes and was independent of socioeconomic status and lifestyle factors. 

With reference to specific psychological conditions, the presence of depression and/or anxiety has been demonstrated to be an independent risk factor for developing chronic physical conditions [8]. 

The prevalence of depression in patients with a myocardial infarction in hospital and post-discharge is estimated to be 15% to 20% [9,10]. According to the available literature, depressive symptoms, and even more major depression, seem to be one of the strongest specific psychological determinants for developing cardiovascular diseases and with the worst outcomes [11,12]. Anxiety and hostility are also associated not only with increased cardiac morbidity in healthy people but also poor prognosis in patients affected by chronic heart diseases [13]. Multidisciplinary cardiac rehabilitation (CR) is a multidisciplinary systematic approach designed to apply secondary prevention therapies of known benefit, inclusive of medical evaluation, prescriptive exercise, cardiac risk factor modification, education, counselling, and behavioral interventions [14,15]. CR could be a proper time to identify emotional disorders or psychological distress and define a specific psychological profile to help patients to cope with stressful events in order to improve their outcomes [16]. But still, to the best of our knowledge, there is a lack of evidence on how psychological distress affects the immediate cardiovascular performance during multidisciplinary rehabilitation programs. On the other hand, we can speculate that psychological distress can influence stress response after surgery and it can influence prognosis, including survival. For instance, in pediatric surgery, it has been demonstrated how animal-assisted therapy can facilitate the rapid recovery of vigilance and activity after anesthesia, modifying pain perception and inducing emotional prefrontal responses. An adaptative cardiovascular response has also been demonstrated [17].

The hypothalamic–pituitary–adrenal (HPA) axis is one of the significant pathways targeted by the stress response. High cortisol levels due to psychological distress can affect several body functions, including heart function, being a known risk factor for developing cardiovascular diseases [18]. Psychological distress may be caused by a stressful life event, such as grief, which could also be associated with other psychological issues and personality characteristics, which are usually not sufficient for an actual psychiatric diagnosis. It has been reported that adverse events in environmental, family, and social contexts affect the development or exacerbation of pathological conditions, especially in childhood, making individuals more vulnerable to psychological distress and to developing diseases during adolescence and young adulthood [19,20]. 

The aim of this study was to describe and analyze how psychological distress can influence cardiovascular performance in patients after cardiac surgery undergoing cardiac rehabilitation. Furthermore, considering that the reaction to stress takes place in a translational sense between the stressful event and the characteristics of the subject, our goal was to evaluate not only the psychological profile but also the psychological distress and the level of perceived stress, to better describe the impact of these aspects on cardiovascular performances.

## 2. Materials and Methods

### 2.1. Study Population 

This is an observational study conducted on data produced in a naturalistic setting. Patients were referred to cardiac rehabilitation as part of routine clinical practice, not specifically to conduct the study. All patients at the time of admission to hospital signed the statement of agreement to all the necessary procedures and treatments The study sample included 57 patients (N = 57), 33 men (57.9%) and 24 women (42.1%), with a mean age of 63.8 years. Participants were patients admitted to the Department of Cardiological Rehabilitation of the Fondazione Policlinico Gemelli IRCCS (Rome, Italy) after cardiac surgery for valvular or coronary disease. The population was equally distributed between patients undergoing valvular (53%) and coronary surgery (47%). The patients, after undergoing cardiac surgery, initiated the rehabilitation process. They were not eligible to enter the program if there was the presence of a known history of psychiatric disorders, presence of post-surgery cardiovascular complications, absence of heart failure, presence of infective post-surgery complications, or presence of physical limitations that prevented the patient from performing cardiovascular functional evaluation and, thus, the exercise-based rehabilitation program. The psychological assessment was carried out within 5 days of hospital admission.

### 2.2. Cardiovascular Assessment and Rehabilitation Program

At the admission and at the end of the CR program, cardiovascular performance was tested by a 6 min walking test (6MWT), a submaximal exercise test used to assess functional physical capacity by measuring the meters walked by the patients on a flat walking path in six minutes. The patient’s heart rate, blood pressure, and oxygen saturation were measured before and after exercise. 

Maximum oxygen uptake (VO2 Max) is the maximum amount of oxygen a person can absorb from inspired air while performing maximal dynamic exercise; it is considered the best measure of cardiovascular fitness and exercise capacity [21]. The cardio-pulmonary test (CPET) with the measurement of peak VO2 is the reference standard for the assessment of aerobic capacity [22]. Since it is not feasible to carry out an assessment via CPET, the LP Cahalin et al. formula [23] was used to obtain the VO2 Max values: Peak VO2 Max = 0.03 × distance (m) + 3.98. 

The intensity of physical exercise perceived by the patients during the cardiological rehabilitation program was quantified through the subjective evaluation system of perceived effort (BORG CR-10 scale); this scale is based on subjective physical sensations during physical exertion, such as increased heart rate, respiratory rate, increased sweating, and muscle fatigue. The evaluation system involves the use of a scale numbered in ascending order from 0 to 10, where 0 corresponds to the minimum effort and 10 to the maximum perceived effort. 

The correlation between the 6MWT and peak VO2 was further studied by Roul et al. [24], who demonstrated that there is a correlation between the two parameters which is more significant the more that the cardiac function is compromised. In this sense, the 6MWT assumes the meaning of maximal test, especially in subjects with a daily activity relatively close to their maximum exercise capacity, as happens in most patients who access the cardiac rehabilitation service. The prognostic value of the 6MWT was first reported by Bittner et al. [25]; furthermore, Cahalin et al. [23] concluded that in patients with severe cardiac impairment, the 6 min time interval predicted short-term event-free survival with a good approximation, while peak VO2 could better predict survival global and free from long-term events.

In relation to the results obtained during the first cardiovascular assessment, the patients were directed towards an in-hospital exercise program with gradual increase, which included the following, in order of progressive implementation: pedal board; exercise bike; treadmills. We started with physical activity lasting 15–20 min, until reaching an average duration of 45–50 min. The increase in the intensity of the exercise or the transition to a type that involved greater aerobic commitment was based on the detection of an adequate chronotropic response compared to what was expected. If hypotension or other disturbances occurred during physical activity, patients reduced or maintained the intensity of exercise performed on the previous day.

### 2.3. Symptom Checklist-90-Revised

As part of the multidisciplinary clinical practice in cardiac rehabilitation, within 5 days of admission, each patient underwent the Italian version of the Symptom Checklist-90-Revised (SCL-90-R) [26], a self-report questionnaire, to assess the intensity of psychological symptoms that occurred during the last week prior to administration. The SCL-90-R includes 90 questions to be answered on a 5-point Likert scale (0: not at all; 4 a lot). The questions are combined in nine primary symptomatologic scales, which specifically are about the following: somatic complaints (Somatization), obsessive–compulsive behaviors (Obsessive–Compulsive); feelings of inappropriateness (Interpersonal Sensitivity); depressive symptoms (Depression); general signs of anxiety (Anxiety); anger–aggression related behaviors (Hostility); phobic symptoms (Phobic anxiety); paranoid ideation symptoms (Paranoid ideation); and Psychoticism. There are also additional items that combine sleep and eating disorders together (Other problems). These 10-dimensional scales lead to three global indices: The Global Score Index (GSI) indicates the intensity of the symptoms perceived; the Positive Symptom Total (PST) indicates the number of symptoms reported in the completion of questionnaire; and the Positive Symptom Distress Index (PSDI) indicates the amplitude of symptom distress. Cut-off scores in the Italian general population are available for this instrument.

The SCL-90-R can reflect the configuration of psychological symptoms of both non-clinical and clinical subjects, and it can also be used with adolescents (from 13 years old). The presence of an operator is maintained to ensure that subjects understand the most difficult items. Even if a clinical examination is fundamental, this assessment instrument is able to identify psychiatric and psychological symptomatology. In clinical practice, it can be used once to evaluate the subject’s level of general discomfort, or repeatedly to obtain more detailed and specific profiles, documenting formal results, response trends, or for pre- and post-treatment evaluation. The SCL-90-R is also used as an outcome measurement in psychotherapy research and in primary care settings [26]. The SCL-90-R assesses the subjective experience of psychopathology; thus, it is necessary to compare the prevalence of disturbances when the same tool is applied. Unfortunately, there is a lack of studies assessing the prevalence of psychopathology using the SCL-90-R in the elderly population without a diagnosis of a psychiatric condition. We referred to the study by Larsen et al. that reported the prevalence of significant psychopathology among individuals aged >18 years [27]. 

### 2.4. General Health Questionnaire

To assess levels of perceived well-being and psychological distress, the 12-item General Health Questionnaire (GHQ-12) [28] was administered. It is a self-report questionnaire on a 4-point Likert scale (0: more than usual; 3 much less than usual) of symptoms that occurred during the two weeks prior to administration.

### 2.5. Statistics

The change in cardiovascular performance during the rehabilitation period was quantified as the variation in the 6MWT, VO2, and BORG between the admission and the end of the treatment. The significance of the change between baseline and discharge was assessed using a paired-samples *t*-test. 

The correlations between scores obtained on the SCL-90-R and GHQ and the indices of cardiovascular performance (6MWT, VO2, and BORG) were assessed by determining the Spearman’s rho.

## 3. Results 

### 3.1. Cardiovascular Performances 

The 6MWT was performed at admission and repeated at the end of the cardiac rehabilitation program. The test was well tolerated by all patients. At baseline, the mean walking distance in the overall population was 217.1 m; the mean walking distance was 398.2 m when repeated at the end of CR program. The mean VO2 max in the overall population was 10.5 at baseline; the mean VO2 max was 15.9 at discharge. The BORG mean value at baseline was 4.2 while the mean value in the overall population at the end of the cardiovascular rehabilitation was 2.5. Table 1 shows the mean values of admission and discharge 6MWT, VO2 max, and BORG. As shown, a significant improvement was observed in all the scales. 

### 3.2. Prevalence of Pathological Profiles on SCL-90-R and GHQ-12

**Figure 1:**
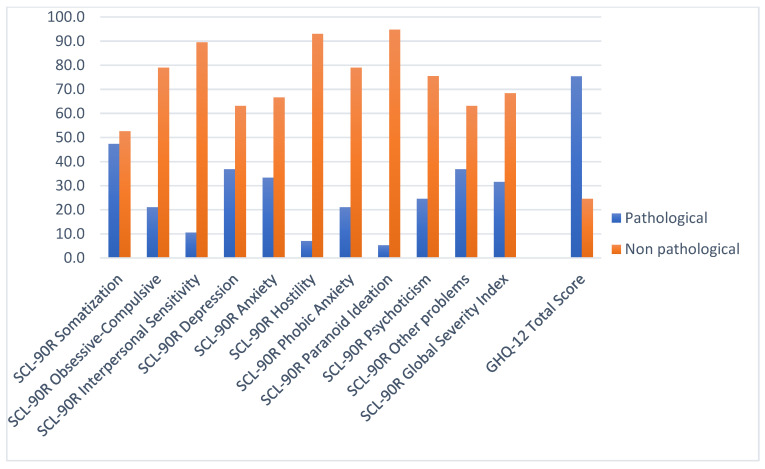
Percentage of patients who scored above (i.e., pathological) and below (i.e., non-pathological) the cut-off scores on the symptom scales of the Symptom Checklist-90 and at the General Health Questionnaire.

**Figure 2:**
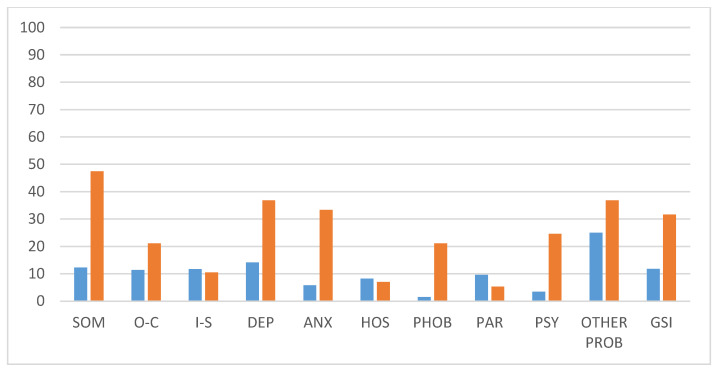
Shows the comparison of the rates of each psychological distress in Symptom Checklist-90 R among individuals aged >18 years (in blue) and in our sample (in orange).

Table 2 and Figure 1 show the distribution of the results obtained by the sample in each of the symptom scales. As shown in Figure 2, for the SCL-90-R score, the prevalence of most of the symptoms in our sample did exceed the general population of healthy adults, as reported in the study by Larsen [27]. Somatic symptoms (47.4%), depressive symptoms (36.8%), and anxiety symptoms (33.3%) were significantly increased. Moreover, symptoms of phobic anxiety (21.1%) and psychoticism (24.6%) were over-represented. The GSI was above the threshold in 31.6% of the patients. As for the GHQ-12, 75.4% of the sample reported an abnormally negative perception of their health status. 

### 3.3. Correlations between Cardiovascular Performance and Psychological Measures

Table 3, Table 4 and Table 5 display the correlations between the variations in cardiovascular performance indices and psychological measures.

As shown in Table 3, we observed an inverse correlation between the variation in the 6MWT and depression (*p* = 0.48) according to the SCL-90-R. Moreover, the Positive Symptom Distress Index (PSDI) (*p* = 0.022) of the SCL-90-R showed an inverse correlation with the variation in 6MWT. We also observed a significant inverse correlation between GHQ-12 (*p* = 0.040) and 6MWT.

Similar results were obtained as for the variation in VO2 max (Table 4). This parameter was inversely correlated with PSDI (*p* = 0.023) and GHQ-12 (*p* = 0.041). We did not observe a significant inverse correlation with any of the primary symptomatologic scales of the SCL-90-R. 

Finally, as shown in Table 5, only hostility showed an inverse correlation with the variation in the BORG (*p* = 0.033). We did not observe any other statistically significant correlations between psychologic measures and BORG variation.

## 4. Discussion

It has been observed how preoperative psychological preparation has positive effects on postoperative outcomes [28]. This is one of the first studies assessing the psychological profile of a cohort of patients entering an intensive in-hospital cardiac rehabilitation program after cardiac surgery [29]. According to the results obtained from the SCL-90-R scores, somatic symptoms, and depressive and anxiety symptoms were over-represented in our sample; such evidence suggests a possible role of these psychological profiles in the development of cardiovascular disease and in the symptoms reported by the subjects under examination, even before the beginning of the rehabilitation program. Remarkably, results on cardiovascular performance, measured by the variations in the 6MWT and VO2 max, have shown to be correlated to psychological profile, particularly to the presence of depression and anxiety. These data are in agreement with the available literature, which has amply shown a significant increase in cardiovascular risk—for both the incidence of chronic heart disease in initially healthy individuals and poor prognosis in patients with a previous diagnosis of chronic heart disease—among patients with a psychological profile corresponding to depression and/or anxiety and hostility. For instance, it has been found that depression is highly prevalent in patients with cardiovascular disease, and depression can lead to adverse cardiovascular outcomes [30,31]. With regard to the association between heart function and anxiety, a meta-analysis has shown that individuals with high anxiety were at increased risk of incident coronary heart disease [32]. In addition, while a correlation between somatic symptoms and depression is well-known [33], an association of these symptoms with cardiovascular risk has been reported in a specific population, such as middle-aged women [34]. 

However, it must be considered that the psychological risk is not uniform across all patients and that psychological factors, including stressful life events, might cluster together within individuals, enhancing psychological distress [35]. Previous studies have suggested that, given the possible clustering of several psychological aspects and the subjective response to stress, the cardiotoxicity of psychological distress could relate to the psychological burden, possibly modulated by vulnerability or resilience factors [36,37,38]. In fact, vulnerability and resilience factors are also related to early adverse experiences (ACEs), which can be associated with personality characteristics. ACEs can also compromise subjective psychological status and personal relationships and professional choices throughout a lifetime, in order to cause a vicious cycle and permanent individual psychological distress [19,20]. This condition can have a consequential effect on cardiovascular functions and on rehabilitation results. This hypothesis could be further supported by the fact that the GSI and the GHQ-12 were above the threshold in 31.6% of the patients and in the 75.4% of the sample, respectively, indicating the intensity and the burden of the symptoms perceived. 

Symptoms of phobic anxiety and psychoticism, together with depression, anxiety, and somatic symptoms, are expressions of a patient’s psychological fragility and of their need for a psychological intervention, in order to improve the efficacy of rehabilitation. According to the Human Birth Theory, a new theory of mind, the mind/brain and body are merged since birth, from the reaction to the new extrauterine environment, specifically light, and they keep this connection throughout life [20,39,40,41]. This mind–body unity needs to be considered in medical intervention, especially in critical surgery, such as cardiac surgery. Another novelty of this study resides in the evaluation of the correlation between the psychological profile of post-cardiac surgery CR patients and derived indexes of cardiovascular performance as changed between the beginning and the end of a CR program. We observed an inverse correlation between the variation in the 6MWT and depression according to the SCL-90-R. These data are in line with the available literature, which have already shown the independent role of depression in predicting the exercise capacity in patients with heart failure [35]. Depression is generally recognized to be a potent risk factor for cardiac, especially coronary, diseases, and its incidence is increasing over time. It is a mental illness, which can be hidden behind psychological distress and affects particularly women. It has been reported that mental stress and emotional arousal can act as triggers of myocardial infarction and other adverse cardiovascular outcomes, so mental stress-induced myocardial ischemia (MSIMI) has been described. Depression and being a woman younger than 50 years may increase the relative risk of MSIMI [42]. Depressive patients do not follow a healthy lifestyle, usually smoke, have eating disorders, are mostly sedentary, and may inconstantly adhere to drug prescriptions. Thus, this profile may predispose or facilitate the occurrence of coronary heart disease or may impair recovery after an acute cardiac event. In the context of CR, depressive patients have trouble in adhering to the prescribed personalized rehabilitation program, and, furthermore, they present symptoms that might limit their exercise-based program. This can be a possible explanation for reduced achievement in parameters related to physical performance. Nevertheless, the results of this study specifically encourage the pursuit of the implementation of psychological evaluation and intervention throughout the in-hospital rehabilitation program. It is of note that this intervention could consist of a psychological support in the CR unit, as a preparative phase to a structured psychotherapy intervention, which can be eventually programmed after discharge, in order to have more stable and longstanding mental and cardiac “healing”.

None of the primary symptom scales in the SCL-90-R showed a significant correlation with the VO2 max variation. Moreover, the overall PSDI and GHQ-12 scores showed an inverse correlation with both the variation in 6MWT and the variation in VO2 max, suggesting that the level of perceived stress plays a decisive role in cardiovascular performance, regardless of the recognition of specific symptom profiles or psychological diagnoses. As already described by previous literature [16,36,37], the same event may be experienced differently by different people due to the heterogeneity of perceived stress, and this may be associated with different biological and psychological consequences. Considering these findings in association with the evidence in the literature, it could be reasonable to evaluate the psychological burden with PSDI and GHQ-12 in all patients undergoing a psychological assessment by means of SCL-90-R at admission in cardiac rehabilitation programs, in order to intercept the complex mechanisms of psychological cardiotoxicity in a timely manner and implement strategies of secondary/tertiary prevention.

Finally, an inverse correlation was observed between the BORG and the SCL-90-R hostility scale; it seems reasonable to suppose that patients bearing this psychological profile have difficulty in accepting a collaborative rehabilitation process and, in parallel, experience a greater burden of their isolated effort. Moreover, these findings are in line with the observation that hostility has been associated with the development of metabolic syndrome [43], the development and the worsening of atherosclerosis [44,45], and poor prognosis in patients affected by chronic heart disease [13].

Overall, the remarkable number of patients with psychological symptoms entering a cardiac rehabilitation program, having an impact on its performance outcomes, reinforces the idea that mental health is a crucial issue in granting and preserving physical, particularly cardiac, health. These findings highlight the need to go into detail regarding the complex relationship between psychological distress and cardiovascular diseases and enhances the need for the early identification of psychological burden in order to implement early and effective interventions. This can show how integrative work with cardiology and psychiatry or psychology units may be crucial for the efficacy of cardiologic intervention and, in particular, of cardiac surgery and rehabilitation after it. 

## 5. Conclusions

Cardiac conditions requiring hospital rehabilitation are associated with increased psychological discomfort, as shown by specific symptoms, in comparison with the general population. During the rehabilitation process, psychological distress significantly affects physical recovery, showing a strong mind–body connection. Cardiac rehabilitation appears to be a proper time to manage psychological distress, in order to help the patients to limit the deleterious effects of stress. Such first evidence urges the deepening of this psychosomatic inter-relationship, to recognize unmet needs and improve specific strategies in rehabilitation, in order to improve cardiac outcomes and reduce adverse consequences. Further studies are necessary to examine the impact of psychological intervention on cardiac outcomes in patients undergoing cardiac rehabilitation.

## Figures and Tables

**Table 1:** Scores obtained by the subjects at admission and at discharge from the rehabilitation unit. |t|: paired-samples *t*-test.

	Mean	SD	|t|	*p*
6MWT baseline	217.1	46.22		
6MWT final	398.2	97.53	15.836	<0.001
VO2 Max baseline	10.5	1.39		
VO2 Max final	15.9	2.92	15.836	<0.001
BORG baseline	4.2	1.95		
BORG final	2.5	1.88	6.082	<0.001

**Table 2:** Percentage of patients who obtained pathological scores on each of the symptomatologic scales of the SCL-90-R and the GHQ-12.

	N. of Subjects with Pathological Score	%
SCL-90-R		
Somatization	27	47.4
Obsessive–compulsive	12	21.1
Interpersonal Sensitivity	6	10.5
Depression	21	36.8
Anxiety	19	33.3
Hostility	4	7.0
Phobic Anxiety	12	21.1
Paranoid Ideation	3	5.3
Psychoticism	14	24.6
Other problems	21	36.8
Global Severity Index	18	31.6
GHQ-12	43	75.4

**Table 3:** Correlations between symptom scale scores of the SCL-90-R and variation in meters walked in the 6 min walking test (6MWT). * indicates the statistically significative values.

	6MWT Variation	
	rho	*p*
Somatization	−0.204	0.128
Obsessive–compulsive	−0.208	0.120
Interpersonal Sensitivity	0.016	0.907
Depression	−0.263 *	**0.048**
Anxiety	−0.032	0.811
Hostility	0.032	0.814
Phobic Anxiety	−0.142	0.291
Paranoid Ideation	−0.081	0.550
Psychoticism	−0.017	0.902
Other problems	−0.221	0.099
Global Severity Index (GSI)	−0.159	0.238
Positive Symptoms Total (PST)	−0.136	0.315
Positive Symptoms Distress Index (PSDI)	−0.302 *	**0.022**
General Health Questionnaire-12 (GHQ-12)	−0.272 *	**0.040**

**Table 4:** Correlations between symptom scale scores of the SCL-90-R and variation in the oxygen consumption (VO2 max). * indicates the statistically significative values.

	VO2 Max Variation	
	rho	*p*
Somatization	−0.203	0.131
Obsessive–compulsive	−0.207	0.122
Interpersonal Sensitivity	0.016	0.906
Depression	−0.260	0.051
Anxiety	−0.029	0.828
Hostility	0.033	0.806
Phobic Anxiety	−0.140	0.298
Paranoid Ideation	−0.078	0.564
Psychoticism	−0.014	0.918
Other problems	−0.220	0.099
Global Severity Index (GSI)	−0.156	0.245
Positive Symptoms Total (PST)	−0.133	0.323
Positive Symptoms Distress Index (PSDI)	**−** 0.301 *	**0.023**
General Health Questionnaire-12 (GHQ-12)	**−** 0.272 *	**0.041**

**Table 5:** Correlations between symptom scale scores of the SCL-90-R and variation in the BORG scale. * indicates the statistically significative values.

	BORG Variation	
	rho	*p*
Somatization	0.116	0.389
Obsessive-compulsive	0.071	0.597
Interpersonal Sensitivity	−0.110	0.414
Depression	0.081	0.548
Anxiety	0.054	0.691
Hostility	−0.283 *	**0.033**
Phobic Anxiety	0.114	0.398
Paranoid Ideation	−0.016	0.907
Psychoticism	0.016	0.908
Other problems	0.156	0.246
Global Severity Index (GSI)	0.073	0.590
Positive Symptoms Total (PST)	0.086	0.527
Positive Symptoms Distress Index (PSDI)	−0.130	0.335
General Health Questionnaire-12 (GHQ-12)	−0.105	0.439

## Data Availability

Data are contained within the article.
